# DNA barcoding for the efficient and accurate identification of medicinal polygonati rhizoma in China

**DOI:** 10.1371/journal.pone.0201015

**Published:** 2018-07-18

**Authors:** Jie Jiao, Wenli Huang, Zhenqing Bai, Feng Liu, Cunde Ma, Zongsuo Liang

**Affiliations:** 1 College of Life Science, Northwest A&F University, Yangling, Shaanxi, China; 2 College of Pharmacy, Shaanxi University of Chinese Medicine, Xianyang, Shaanxi, China; 3 College of Life Science, Yan'an University, Yan'an, Shaanxi, China; 4 Research Department, Buchang Pharma, Xi’an, Shaanxi, China; 5 College of Life Science, Zhejiang SCI-TECH University, Hangzhou, Zhejiang, China; National Cheng Kung University, TAIWAN

## Abstract

Polygonati rhizoma (PR), a traditional medicinal and edible product with various bioactive components (*Polygonatum* polysaccharides, saponins, phenols, and flavonoids), is widely consumed in China. However, other species with morphological characteristics similar to those of the actual components are being used to replace or adulterate PR, causing issues with quality control and product safety. The morphological similarity of PR and its substitutes makes classic morphological identification challenging. To address this issue, DNA barcoding-based identification using *ITS2* and *psbA-trnH* sequences was applied in this study to evaluate the efficiency and accuracy of this approach in identifying PR samples collected from 39 different regions in China. The identification of PR by this method was confirmed by other methods (phylogeny-based and character-based methods), and all the samples were clearly and accurately distinguished. This study highlights the efficient and accurate nature of DNA barcoding in PR identification. Applying this technique will provide a means to differentiate PR from other altered formulations, thus improving product quality and safety for consumers of PR and its products.

## Introduction

Polygonati rhizoma (PR) is a medicinal and edible product recognized by the China Food and Drug Administration. PR has been used during famine [1] and by Chinese Taoists and Buddhists for sustenance during extended fasting [2]. PR is composed of the dry rhizomes of *Polygonatum sibiricum* F. Delaroche, *Polygonatum cyrtonema* Hua, and *Polygonatum kingianum* Coll. et Hemsl., all of which are perennial herbs belonging to the Asparagaceae family [3]. These three species contain multiple bioactive components, such as *Polygonatum* polysaccharides [4], saponins [5], phenols [6], and flavonoids [7], which have various biological functions. Records in the *Compendium of Materia Medica* [8] describe PR as sweet and non-toxic. In China, people use PR as both a healthcare product and a food ingredient in daily meals. It appears to tonify the spleen and kidney, moisten the lungs, and quench thirst [9], according to the primary lung, kidney, and spleen channel classifications in traditional Chinese medicine theories. Moreover, modern pharmacological studies have shown that PR improves immunity [10] and memory [11]; reduces blood glucose [12] and fat levels [13]; and elicits antibacterial [14], antiviral [15], anticancer [16], and anti-aging [17] effects. As it has important edible value in both raw and processed forms, PR has been used to make various foods, such as cakes, biscuits, succade, preserved fruit, pastes, bread, tea, drinks, and wine, as well as products like soaps and shampoos.

Unfortunately, numerous rhizome species with similar morphological traits distributed in the southwest region of China are misused as PR components. These include the rhizomes of *Polygonatum filipes* Merr., *Polygonatum punctatum*, *Polygonatum cathcartii* Baker, *Polygonatum verticillatum* (L.) All., *Disporopsis longifolia*, and *Gelsemium elegans* (Gardn. & Champ.) Benth. Thus, efficiently and accurately distinguishing PR from other rhizome species is essential to assure product quality and consumer safety. The components of PR have particular characteristics, including time-consuming methods for breaking the dormancy [18] and growing the seedlings [19], as well as variable growth environments, all of which make classical identification techniques using herb morphology and biochemistry inefficient and often inaccurate. Therefore, other methodologies are required to effectively identify PR components and distinguish them from non-PR herbs. Molecular identification based on DNA barcoding is a recent tool that has been used to conduct species-level identification [20]. As an effective complement to traditional identification methods, DNA barcoding identification classifies species based on standardized, relatively short DNA sequences, which differ among species and are consistent regardless of environment. To date, DNA barcoding has been used to identify various plant species, such as lichens [21], fungi [22, 23], weeds [24], trees [25–28], and economically important plants such as crops [29] and medicinal and aromatic plants [30–35]. The DNA barcoding system and principles established by Chen [36] can be used to clearly identify Chinese medicinal materials found in various forms, including multi-component medicines, medicinal powders [37] and fragments [38], and the original herb [39]. This system uses the *ITS2* and *psbA-trnH* sequences as the main and auxiliary sequences, respectively, and has successfully identified *Dendrobium* [40], Polygonaceae [41], Rosaceae [42], Araceae [43], and Fabaceae [44]. However, this DNA barcoding system has not been used to identify the components of PR.

In this study, we applied a DNA barcoding system with *ITS2* and *psbA-trnH* sequences to identify PR samples collected from southern China. To validate this DNA barcoding-based method, identification using phylogeny-based and character-based methods was also conducted. To our knowledge, this is the first report demonstrating the use of this *ITS2*/*psbA-trnH*-based DNA barcoding system to efficiently and accurately identify PR. Importantly, our findings have immediate practical implications on the application of DNA barcoding to molecularly identify herbal medicines irrespective of their material state.

## Materials and methods

### Plant materials

In the present investigation, 39 samples were collected from different regions in 11 different provinces of China (Tables 1 and 2). Approximately 100 rhizomes were selected from each region and were maintained in PR GAP base (Buchang Pharma, Lveyang, Shaanxi, China).

**Table 1:** Details of the sampling areas of polygonati rhizoma from different regions in China.

Provinces	Locations’ name	Geographic coordinates	Accession no.	Putative Species	NCBI ID (Top percent identity)	Accession no. (GENE BANK)
Anhui	Chizhou Dajianshan	117°36′32″E 30°39′36″N	S1	*Polygonatum cyrtonema*	*Polygonatum cyrtonema* (100)	KJ745884.1
	Luan Longxueshan	116°42′51″E 31°43′27″N	S2	*P*. *cyrtonema*	*P*. *cyrtonema* (100)	KJ745884.1
	Qingyang Qingyuanshan	118°02′6″E 30°41′9″N	S3	*P*. *cyrtonema*	*P*. *cyrtonema* (100)	KJ745884.1
	Jingxian Qinglongshan	118°33′14″E 30°32′35″N	S4	*P*. *cyrtonema*	*P*. *cyrtonema* (100)	KJ745884.1
	Huangshan	118°10′11″E 30°07′55″N	S5	*P*. *cyrtonema*	*P*. *cyrtonema* (100)	KJ745884.1
Chongqing	Wulong Xiannvshan	107°47′56″E 29°25′38″N	S6	*P*. *cyrtonema*	*Polygonatum kingianum* (100)	KJ745828.1
	Qijiang Huanggaoshan	106°54′23″E 28°58′38″N	S7	*P*. *cyrtonema*	*P*. *kingianum* (100)	KJ745828.1
Fujian	Zhenghe Jinpingcun	119°06′13″E 27°24′54″N	S8	*P*. *cyrtonema*	*Polygonatum curvistylum* (99) *Polygonatum cirrhifolium* (99) *Polygonatum franchetii* (99) *Polygonatum prattii* (99)	KJ745774.1 KJ745802.1 KJ745833.1 KJ745837.1
Guangdong	Shaoguan Longdoushan	113°55′33″E 24°43′21″N	S9	*P*. *cyrtonema*	*P*. *curvistylum* (100) *P*. *cirrhifolium* (100)	KJ745774.1 KJ745802.1
	Qingyuan Dawangshan	113°22′34″E 23°44′4″N	S10	*P*. *cyrtonema*	*P*. *curvistylum* (99) *P*. *cirrhifolium* (99) *P*. *franchetii* (99)	KJ745774.1 KJ745802.1 KJ745833.1
Guangxi	Chongzuo Shanglongxiang	106°51′8″E 22°24′32″N	S11	*P*. *cyrtonema*	*Disporopsis longifolia* (100)	KJ745836.1
	Hezhou Pingshan	111°26′21″E 24°24′51″N	S12	*P*. *cyrtonema*	*P*. *cyrtonema* (100)	KJ745884.1
	Yizhou Jiulongshan	108°37′42″E 24°28′8″N	S13	*P*. *cyrtonema*	*P*. *cyrtonema* (100)	KJ745884.1
	Baise Jitishan	106°41′29″E 23°55′32″N	S14	*P*. *cyrtonema*	*P*. *kingianum* (100)	KJ745828.1
Guizhou	Tongren Liulongshan	109°16′22″E 27°38′25″N	S15	*P*. *cyrtonema*	*P*. *kingianum* (100)	KJ745828.1
	Zhenyuan Longtoushan	108°25′59″E 27°02′22″N	S16	*P*. *cyrtonema*	*P*. *cyrtonema* (100)	KJ745884.1
	Dejiang Daxishan	108°07′34″E 28°15′59″N	S17	*P*. *cyrtonema*	*P*. *kingianum* (100)	KJ745828.1
	Guiyang Panlongdong	106°28′5″E 26°40′44″N	S18	*P*. *cyrtonema*	*P*. *cyrtonema* (100)	KJ745884.1

**Table 2:** Details of the sampling areas of polygonati rhizoma from different regions in China.

Provinces	Locations’ name	Geographic coordinates	Accession no.	Putative Species	NCBI ID (Top percent identity)	Accession no. (GENE BANK)
Henan	Lushi Wangjiashan	111°05′51″E 34°02′8″N	S19	*Polygonatum sibiricum*	*Polygonatum sibiricum* (100)	KJ745880.1
	Nanzhao Dingjiazhuan	112°22′10″E 33°28′56″N	S20	*P*. *sibiricum*	*P*. *sibiricum* (100)	KJ745880.1
	Lingbao Sihecun	111°05′12″E 34°28′48″N	S21	*P*. *sibiricum*	*P*. *sibiricum* (100)	KJ745880.1
	Songxian Checunzhen	112°05′2″E 33°48′38″N	S22	*P*. *sibiricum*	*P*. *sibiricum* (99)	KJ745880.1
	Songxian Zhonghuangcun	111°59′4″E 34°09′49″N	S23	*P*. *sibiricum*	*P*. *cyrtonema* (100)	KJ745884.1
	Lingbao Sucun	110°58′15″E 34°32′45″N	S24	*P*. *sibiricum*	*P*. *sibiricum* (100)	KJ745880.1
Shaanxi	Lveyang Wulongdong	106°12′30″E 33°30′9″N	S25	*P*. *sibiricum*	*P*. *sibiricum* (100)	KJ745880.1
	Ankang Xiangxidong	109°01′42″E 32°39′34″N	S26	*P*. *sibiricum*	*P*. *sibiricum* (100)	KJ745880.1
Sichuan	Yilong Shizishan	106°16′39″E 31°15′46″N	S27	*P*. *cyrtonema*	*P*. *cyrtonema* (100)	KJ745884.1
Yunan	Baoshan Qingshan	99°15′36″E 25°04′6″N	S28	*P*. *cyrtonema*	*P*. *kingianum* (99)	KJ745828.1
	Honghe Mopanshan	103°17′47″E 23°21′3″N	S29	*Polygonatum kingianum*	*P*. *kingianum* (100)	KJ745828.1
	Dali Yuntaishan	100°17′47″E 25°33′58″N	S30	*P*. *cyrtonema*	*P*. *cyrtonema* (99)	KJ745884.1
	Mengzi Gaojiacun	103°23′4″E 23°24′19″N	S31	*P*. *kingianum*	*P*. *kingianum* (100)	KJ745828.1
	Mengzi Jixinshan	103°20′42″E 23°25′42″N	S32	*P*. *cyrtonema*	*P*. *cyrtonema* (100)	KJ745884.1
	Yimen Shizishan	102°08′45″E 24°39′15″N	S33	*P*. *cyrtonema*	*P*. *kingianum* (100)	KJ745828.1
Zhejiang	Huangyan Darenshan	121°20′14″E 28°38′30″N	S34	*P*. *cyrtonema*	*P*. *cyrtonema* (100)	KJ745884.1
	Xianju Xiaoyaoxia	120°36′43″E 28°41′10″N	S35	*P*. *cyrtonema*	*P*. *cyrtonema* (100)	KJ745884.1
	Kaihua Jiujiewu	118°25′33″E 29°07′24″N	S36	*P*. *cyrtonema*	*P*. *cyrtonema* (100)	KJ745884.1
	Tongxiang Longwangmiao	120°28′32″E 30°35′2″N	S37	*P*. *cyrtonema*	*P*. *cyrtonema* (100)	KJ745884.1
	Lishui Huangjiashan	120°04′12″E 28°31′14″N	S38	*P*. *cyrtonema*	*P*. *cyrtonema* (100)	KJ745884.1
	Tiantai Tiantaishan	120°57′56″E 29°08′15″N	S39	*P*. *cyrtonema*	*P*. *cyrtonema* (100)	KJ745884.1

### DNA extraction

Fresh rhizome tissue samples from each region were disinfected with 75% alcohol, frozen in liquid nitrogen, and preserved at −80°C. Total genomic DNA was extracted following the Doyle and Doyle method with little modification [45]. DNA was isolated from 0.5 g of rhizome tissue. Purified total DNA was quantified using a NanoDrop 2000 instrument (Thermo Fisher Scientific, Waltham, MA, USA). The DNA samples were then diluted to 30 ng/μL and stored at −20°C until *ITS2* and *psbA-trnH* analysis.

### *ITS2* and *psbA-trnH* screening and amplification

Previously published *ITS2* and *psbA-trnH* primers [32] were synthesized by Shanghai Sangon Biological Engineering Technology and Services (Shanghai, China). PCR amplification was performed with a Veriti 96-well Thermal Cycler (Applied Biosystems). Amplification reactions were conducted in 20 μL reaction volumes in 1.5 mL microfuge tubes with 10.0 μL of 2× Es Taq MasterMix (CWBIO, China), which contains Taq DNA polymerase, 2× Taq PCR buffer, 3 mM MgCl_2_, and 400 μM dNTP mix, along with 1.0 μL of 1 μM primer, 1.0 μL of 30 ng/μL DNA template, and 7.0 μL ddH_2_O (CWBIO). The PCR amplification procedure was set as follows: an initial denaturation of 5 min at 94°C; 30 cycles of 1 min denaturation at 94°C, 1 min annealing at 55°C, and 1.5 min extension at 72°C; and a final extension for 7 min at 72°C. All amplification products were separated on a 1.5% agarose gel using 1× TBE buffer by electrophoresis. The gel was stained with ethidium bromide and visualized with a gel documentation system (Bio-Rad Universal Hood II).

### Data collection and analysis

PCR amplified products with high reproducibility and a clear single target band were recovered, and each product was sequenced by Shanghai Sangon Biological Engineering Technology and Services using a bidirectional sequencing method with the amplification primers. Sequences were proofread and spliced with DNAMAN 5.0 software, and the low-quality sequences and primer regions were removed. We blasted each sequence using NCBI BLAST software, and the top search hit was used as the reference sequence. Multiple sequence alignment using the ClustalW program, variable site analysis using the Data Explorer program, and genetic distance (GD) calculations using the Find Best DNA Models program were all conducted with MEGA 6.06 software. We used the nearest distance method to determine the “best close match” for species identification, using 95% intraspecific distance as the threshold [46]. Finally, a neighbor-joining (NJ) tree, which is closely related to the homology of the sequences, was estimated with the model selected by MEGA 6.06 using the GDs between the sampled sequences and the reference sequences calculated by the Compute Pairwise Distance program. A total of 1,000 bootstrap replicates were chosen to test the phylogeny for species identification. To further test the NJ tree, a maximum-likelihood (ML) tree was also estimated with the same models.

### Phylogenetic analysis

DNA barcoding sequences of species in *Polygonatum* and other outgroups (*Heteropolygonatum*, *Asparagus*, and Zingiberaceae) were searched for in NCBI’s GenBank, which includes deposited data from other researchers and institutions. We collected all the sequences in these NCBI BLAST searches and organized them using the initial letter of their specific name as the norm. Two alignments were generated with the sequences within and among species. Sequences were aligned using ClustalW in the MEGA 6.06 software, which first processes the pairwise sequence alignment representing the relationship between them and then conducts multiple sequence alignments using an asymptotic approach. Thus, the GD among species precisely characterizes the genetic relationship among them.

The best-fit model of evolution and an optimal data-partitioning scheme were chosen using the Find Best DNA/Protein Models program in MEGA 6.06, with each codon position being chosen as an *a priori* data subset. ML was used as the statistical method using partial deletion gaps, with 95% site coverage as the cutoff for Gaps/Missing Data Treatment and moderate as the Branch Swap Filter. GDs were calculated using the Compute Pairwise Distances program with the Substitution Model selected and partial deletion gaps with 95% site coverage set as the cutoff. An ML tree was constructed in MEGA 6.06 using the models with the following settings: partitioning scheme selected, “Nearest-neighbor-interchange” chosen as the ML heuristic method, “Make initial tree automatically” as the initial tree for ML, and “Moderate” as the branch swap filter. A total of 1,000 bootstrap replicates were chosen to test the phylogeny illustrating phylogenetic relationships among species. Using the bootstrap values to test the credibility of the evolutionary tree branch, we can predicate its veracity.

### Topology tests

There are two types of errors in phylogenetic trees: topological errors and branch length errors. Topological difference tests are needed to determine tree reliability, and branch length errors can also be tested using bootstrap tests. When the number of sequences is large and the extent of sequence divergence is low, it is generally difficult to reconstruct the true tree by any method. However, the bootstrap consensus tree often gives a reasonably good tree. Although weakly supported interior branches might differ, the bootstrap consensus tree obtained with the NJ method is usually similar to that obtained with the ML method. Therefore, we chose the NJ method to reconstruct the NJ tree and verify the topological tree constructed with the ML method.

### Character-based tests

According to our BLAST searches, *Polygonatum* species have low *psbA-trnH* diversity. This makes distance-based identification more challenging, because a query sequence can have a nearly identical distance to multiple, different reference sequences. To circumvent this issue, we used the previously published character-based identification key named “characteristic attributes” (CAs) [47], which consists of 14 nucleotide characters (Tables 3 and 4) at specific positions across the *psbA-trnH* barcoding region that were used to identify PR species. This CA system was developed from an alignment of 32 reference sequences with diagnostic states that are specific to each PR species.

**Table 3:** Character based identification for samples.

Menu listing	Character positions (22, 27, 65, 67, 103, 104,125, 127, 128, 129, 130, 132, 172, 429)	Species identified
S1	CGCGTCGTTTCTTA	*Polygonatum cyrtonema*
S2	CGCGTCGTTTCTTA	*Polygonatum cyrtonema*
S3	CGCGTCGTTTCTTA	*Polygonatum cyrtonema*
S4	CGCGTCGTTTCTTA	*Polygonatum cyrtonema*
S5	CGCGTCGTTTCTTA	*Polygonatum cyrtonema*
S6	CGCGCTAGAAACTC	*Polygonatum kingianum*
S7	CGCGCTAGAAACTC	*Polygonatum kingianum*
S8	CGCACTGTTTCTTA	Incomplete certain (multi-species)
S9	CGCACTGTTTCTTA	Incomplete certain (multi-species)
S10	CGCACTGTTTCTTA	Incomplete certain (multi-species)
S11	CGCGTCGTTTCTTC	Non-retrieved
S12	CGCGTCGTTTCTTA	*Polygonatum cyrtonema*
S13	CGCGTCGTTTCTTA	*Polygonatum cyrtonema*
S14	CGCGCTAGAAACTC	*Polygonatum kingianum*
S15	CGCGCTAGAAACTC	*Polygonatum kingianum*
S16	CGCGTCGTTTCTTA	*Polygonatum cyrtonema*
S17	CGCGCTAGAAACTC	*Polygonatum kingianum*
S18	CGCGTCGTTTCTTA	*Polygonatum cyrtonema*
S19	CGCGTCGTTTCTCA	*Polygonatum sibiricum*
S20	CGCGTCGTTTCTCA	*Polygonatum sibiricum*
S21	CGCGTCGTTTCTTA	*Polygonatum cyrtonema*
S22	CGCGTCGTTTCTCC	Non-retrieved

**Table 4:** Character based identification for samples.

Menu listing	Character positions (22, 27, 65, 67, 103, 104,125, 127, 128, 129, 130, 132, 172, 429)	Species identified
S23	CGCGTCGTTTCTTA	*Polygonatum cyrtonema*
S24	CGCGTCGTTTCTCA	*Polygonatum sibiricum*
S25	CGCGTCGTTTCTCA	*Polygonatum sibiricum*
S26	CGCGTCGTTTCTCA	*Polygonatum sibiricum*
S27	CGCGTCGTTTCTTA	*Polygonatum cyrtonema*
S28	CGCGCTAGAAACTC	*Polygonatum kingianum*
S29	CGCGCTAGAAACTC	*Polygonatum kingianum*
S30	CGCGTCGTTTCTTA	*Polygonatum cyrtonema*
S31	CGCGCTAGAAACTC	*Polygonatum kingianum*
S32	CGCGTCGTTTCTTA	*Polygonatum cyrtonema*
S33	CGCGCTAGAAACTC	*Polygonatum kingianum*
S34	CGCGTCGTTTCTTA	*Polygonatum cyrtonema*
S35	CGCGTCGTTTCTTA	*Polygonatum cyrtonema*
S36	CGCGTCGTTTCTTA	*Polygonatum cyrtonema*
S37	CGCGTCGTTTCTTA	*Polygonatum cyrtonema*
S38	CGCGTCGTTTCTTA	*Polygonatum cyrtonema*
S39	CGCGTCGTTTCTTA	*Polygonatum cyrtonema*

## Results

### Sequence amplification, data collation, and preliminary analysis

**Fig 1:**
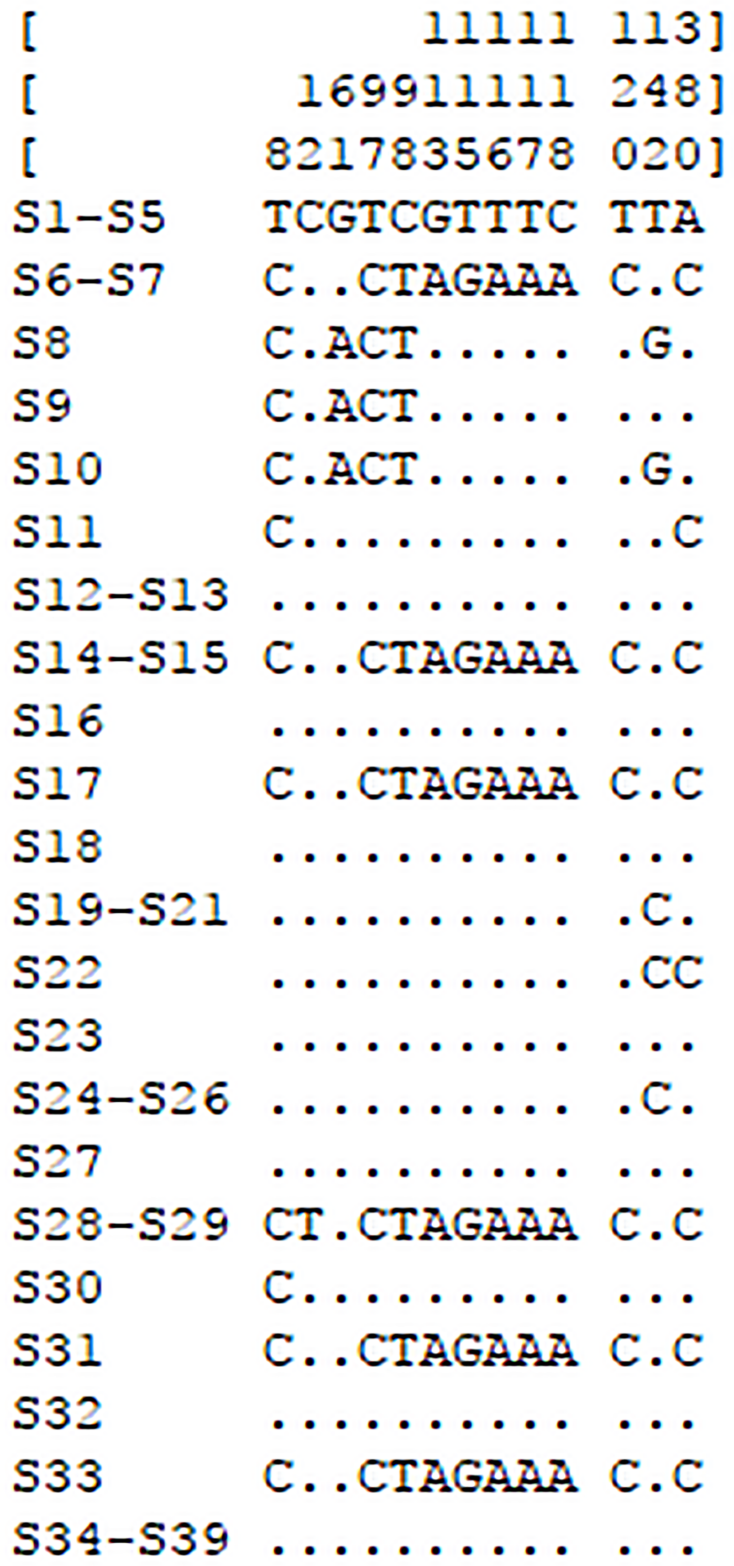
Variation sites of *psbA-trnH* sequences of samples.

In our analysis using *ITS2* and *psbA-trnH* primers, *ITS2* sequences with polymorphic bands (S1A Fig) were not favorable for PR species identification. However, *psbA-trnH* sequences (approximately 650 bp) were clear and formed single bands, which could be used to identify PR species (S1B Fig). After each band was recovered and amplified, the samples were labeled and sequenced. After proofreading, aligning, and removing the low-quality sequences and primer sequences, sequence length varied from 529 to 603 bp, with the G+C and A+T content ranging from 34.8% to 35.6% and 64.4% to 65.2%, respectively (Table 5). All the sequences showed a total of 13 variable sites among the samples in the multiple sequence alignment (Fig 1).

**Table 5:** Sequences length and codon content of *psbA-trnH* sequences.

Regions	T(%)	C(%)	A(%)	G(%)	Length (bp)	Regions	T(%)	C(%)	A(%)	G(%)	Length (bp)
S1	34.7	16.9	30.1	18.3	602	S21	35.0	16.8	30.2	18.0	529
S2	34.7	16.9	30.1	18.3	602	S22	35.0	17.0	30.1	18.0	529
S3	34.7	16.9	30.1	18.3	602	S23	34.7	16.9	30.1	18.3	602
S4	34.7	16.9	30.1	18.3	602	S24	35.0	16.8	30.2	18.0	529
S5	34.7	16.9	30.1	18.3	602	S25	35.0	16.8	30.2	18.0	529
S6	34.0	17.2	30.5	18.2	603	S26	35.0	16.8	30.2	18.0	529
S7	34.0	17.2	30.5	18.2	603	S27	34.7	16.9	30.1	18.3	602
S8	34.4	17.1	30.1	18.4	602	S28	34.0	17.2	30.5	18.2	603
S9	34.7	17.1	30.0	18.2	603	S29	34.0	17.2	30.5	18.2	603
S10	34.4	17.1	30.1	18.4	602	S30	34.6	17.1	30.1	18.3	602
S11	34.5	17.1	29.9	18.5	595	S31	34.0	17.2	30.5	18.2	603
S12	34.5	17.1	29.9	18.5	595	S32	34.7	16.9	30.1	18.3	602
S13	34.5	17.1	29.9	18.5	595	S33	34.0	17.2	30.5	18.2	603
S14	34.5	17.1	29.9	18.5	595	S34	34.7	16.9	30.1	18.3	602
S15	34.7	16.9	30.1	18.3	602	S35	34.7	16.9	30.1	18.3	602
S16	34.7	16.9	30.1	18.3	602	S36	34.7	16.9	30.1	18.3	602
S17	34.7	16.9	30.1	18.3	602	S37	34.7	16.9	30.1	18.3	602
S18	34.7	16.9	30.1	18.3	602	S38	34.7	16.9	30.1	18.3	602
S19	35.0	16.8	30.2	18.0	529	S39	34.7	16.9	30.1	18.3	602
S20	35.0	16.8	30.2	18.0	529						

### DNA barcoding identification

Tables 1 and 2 shows the top hits from the BLAST alignment selected as the reference sequences. Sequences from samples collected from six regions (S19, S20, S21, S24, S25, and S26) were identified as *Polygonatum sibiricum*, sharing 100% identity, while sequences from samples collected from S22 shared 99% identity. Furthermore, sample sequences obtained from 19 regions (S1−S5, S12, S13, S16, S18, S23, S27, S32, S34, and S35−S39) were identified as *P*. *cyrtonema*, and with the exception of S30 (which shared 99% identity), all the sequences shared 100% identity. Sample sequences from nine regions (S6, S7, S14, S15, S17, S28, S29, S31, and S33) are relatively similar to that of *P*. *kingianum*, while the sequence from S11 shared 100% identity with that of *Disporopsis longifolia*. Interestingly, sample sequences from three regions (S8, S9, and S10) shared 99% identity with different reference sequences. Hence, four species were identified in our analysis: *P*. *sibiricum* (7), *P*. *cyrtonema* (19), *P*. *kingianum* (9), and *D*. *longifolia* (1). The other three samples cannot be identified clearly at present.

We used MEGA 6.0 in association with the Tamura 3-parameter model to calculate the GDs within and among sample sequences and reference sequences. GDs between samples ranged from 0.000 to 0.023 (S2 Fig), with 0.000–0.002 indicating being present within species and 0.002–0.023 meaning being present among species. According to study by Meier [48], barcoding gap of sequences is quantified as 0.000, which indicates that barcoding gap does not exist in our samples. *P*. *sibiricum* indicated a close GD with *P*. *cyrtonema* (0.002) and was the farthest from *P*. *kingianum* (0.021–0.023). In addition, *P*. *sibiricum* showed moderate GDs with *P*. *odoratum* (0.004), *P*. *filipes* (0.004), *P*. *curvistylum* (0.010), *P*. *cirrhifolium* (0.010), *P*. *prattii* (0.010), *P*. *franchetii* (0.008), and *D*. *longifolia* (0.006). The GD between *P*. *cyrtonema* and *P*. *kingianum* was 0.019, while the GDs varied for *P*. *cyrtonema* with *P*. *odoratum* (0.002), *P*. *filipes* (0.002), *P*. *curvistylum* (0.008), *P*. *cirrhifolium* (0.008), *P*. *prattii* (0.008), *P*. *franchetii* (0.006), and *D*. *longifolia* (0.004). Among the *Polygonatum* species, *P*. *kingianum* had the farthest GD with *P*. *odoratum* (0.021), followed by *P*. *filipes* (0.021), *P*. *curvistylum* (0.015), *P*. *cirrhifolium* (0.015), *P*. *prattii* (0.015), *P*. *franchetii* (0.013), and *D*. *longifolia* (0.015). Therefore, *P*. *kingianum* has a closer relationship with *P*. *cyrtonema* than with *P*. *sibiricum*. According to the nearest distances method, we verified that sample sequences from 18 regions are classified as *P*. *cyrtonema*; those from nine regions, as *P*. *kingianum*; those from seven regions, as *P*. *sibiricum*; and that from one region, as *D*. *longifolia*. Among these, the sample sequences from S30 had equal GDs with *P*. *cyrtonema* and *D*. *longifolia*, while sample sequences from S8 and S10 had equal GDs with *P*. *curvistylum*, *P*. *cirrhifolium*, and *P*. *prattii*. Sequences from the S9 region also have the same GD with *P*. *curvistylum* and *P*. *cirrhifolium*. Therefore, this analysis using the nearest distances method supports our results from the BLAST analysis, with the exception of sample sequences from the S30 region.

**Fig 2:**
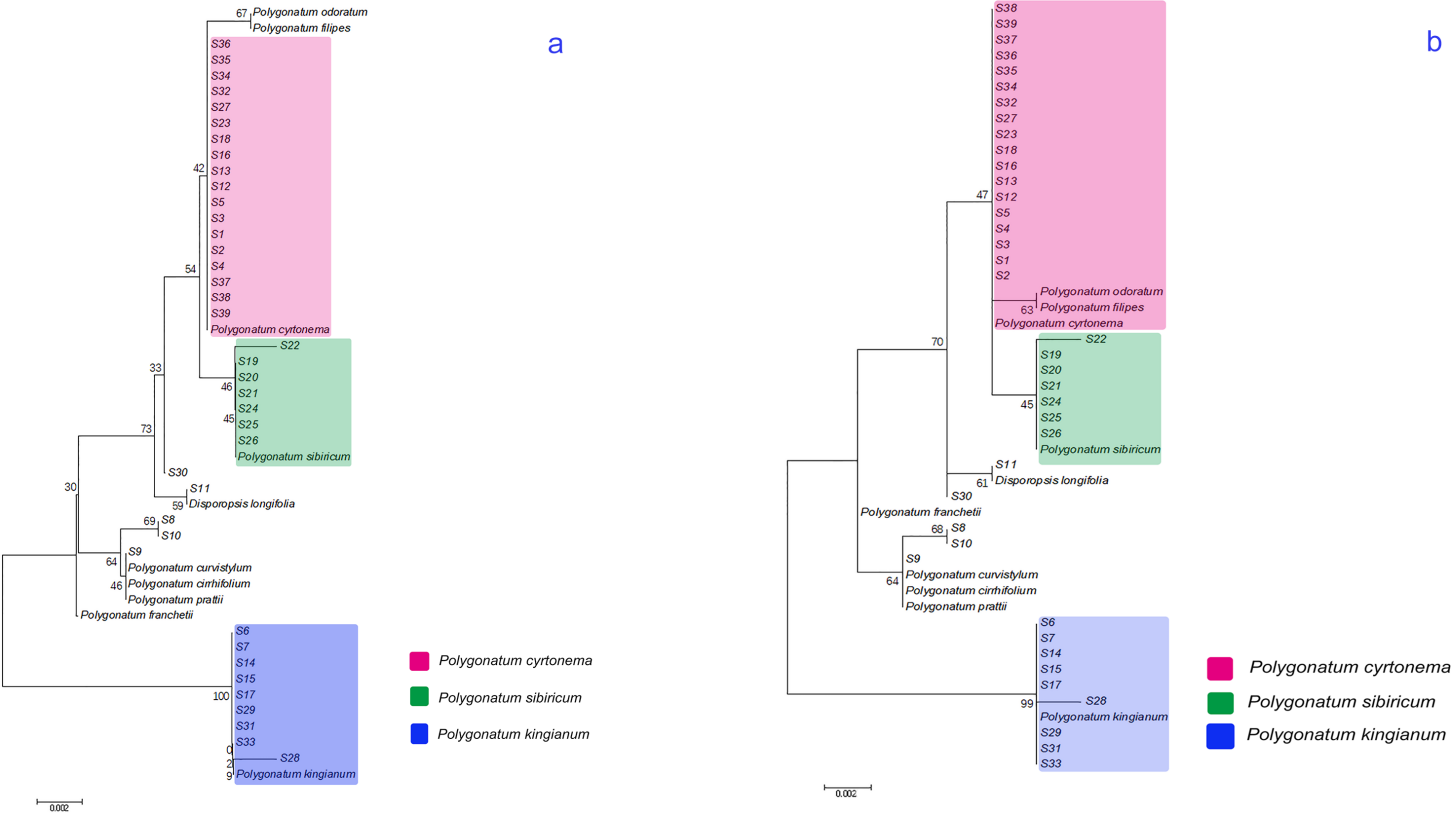
Neighbour-joining tree (a) and maximum likelihood tree (b) constructed based on *psbA-trnH* sequences.

To visualize these results, an NJ tree was generated with the GDs using MEGA 6.06 (Fig 2A). All regions were divided into two clusters with a support rate of 100%. One cluster contained nine regions belonging to *P*. *kingianum*, while another cluster contained the remaining regions. To further verify this NJ tree, an ML tree was also generated (Fig 2B). In this tree, all the sampled regions were clustered into two clusters with a 99% support rate. Notably, the ML tree revealed more accurate genetic relationships compared to the NJ tree.

### Phylogeny-based tests

A total of 207 *psbA-trnH* sequences from *Polygonatum* were downloaded from NCBI’s GenBank. After identical sequences deposited by different researchers were removed using ClustalW, 32 sequences were selected (S1 Table). Through data collection and analysis, we found that the reference sequences of *psbA-trnH* from the same species were not the same in *P*. *cyrtonema* and *P*. *odoratum*. This phenomenon may be related to the different regions of origin. In our sampled sequences, 32 reference sequences and four outgroup sequences, including *D*. *longifolia* (GenBank No.: KJ745836.1), *Heteropolygonatum* (GenBank No.: KJ745790.1), *Asparagus* (GenBank No.: KC704269.1), and Zingiberaceae (GenBank No.: EU552521.1), were used to construct the ML tree.

**Fig 3:**
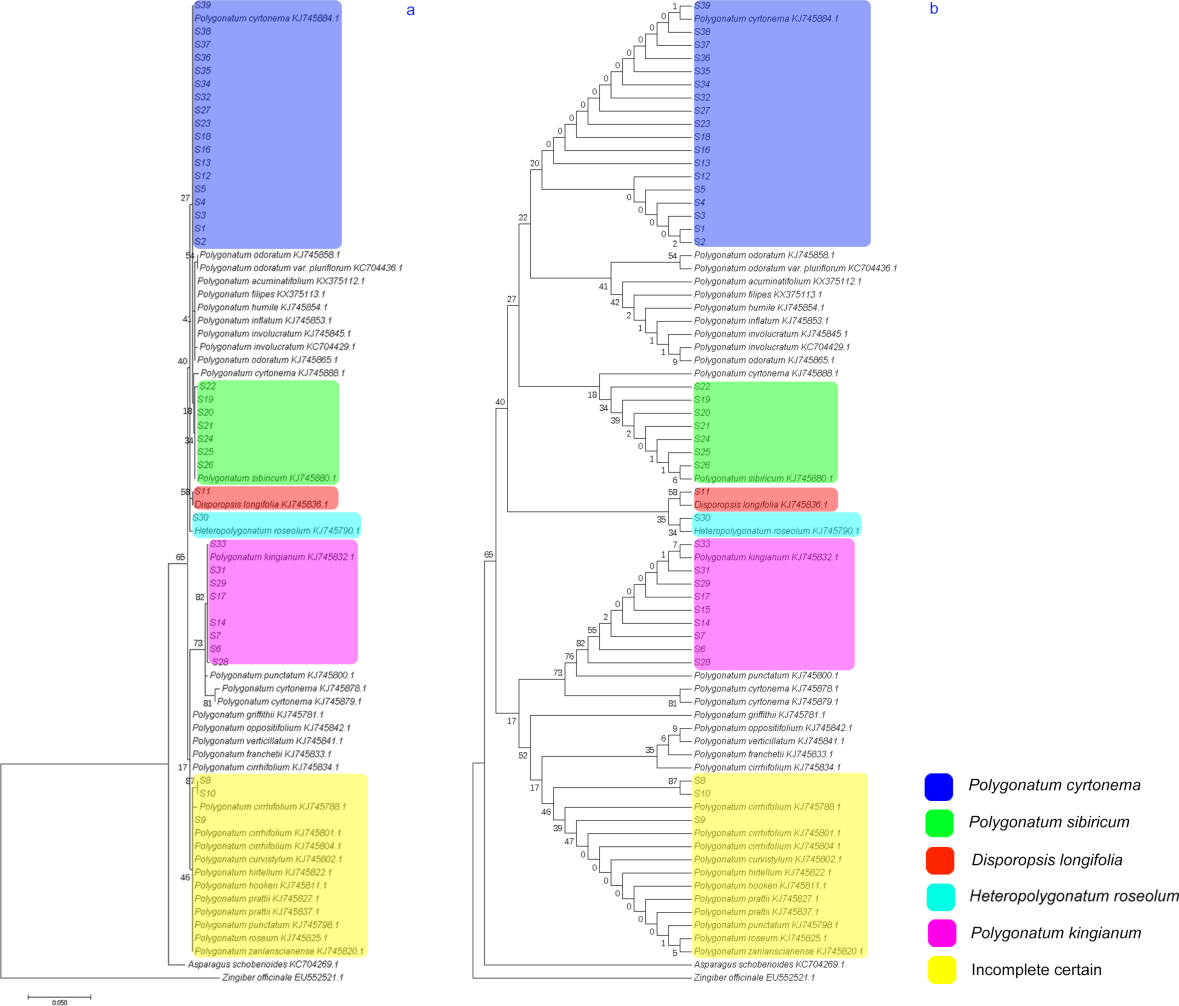
Phylogeny tree (a) and its topology (b) of maximum likelihood tree constructed based on *psbA-trnH* sequences.

The constructed ML phylogeny tree (Fig 3A) indicates that our sample sequences can be classified into six groups, which supports our species identification with the DNA barcoding-based method. In this tree, *psbA-trnH* sequences of some species do not appear to be unique, such as those for *P*. *cyrtonema* (4), *P*. *odoratum* (2), *P*. *cirrhifolium* (4), and *P*. *prattii* (2). The reason for this might stem from similar sequences being uploaded by different researchers at different institutions. This would result in the sequences being slightly different (individual base changes) because of the plants’ different growth environments or small interspecific divergences at the *psbA-trnH* region. By the ML tree method, the samples identified as *P*. *cyrtonema* showed a closer evolutionary relationship with *P*. *cyrtonema*, having a 19% support rate, compared with the DNA barcoding-based method. Samples identified as *P*. *sibiricum* appeared to have a close evolutionary relationship with both *P*. *sibiricum* and *P*. *cyrtonema*, with support rates of 26% and 14%, respectively. Furthermore, the ML tree showed 82% and 79% support rates for evolutionary relationships among the samples identified as *P*. *kingianum*, *P*. *punctatum*, *P*. *cyrtonema*, and *P*. *cyrtonema*. The topology of the ML tree (Fig 3B) also supports the conclusions drawn from our phylogeny tree.

Finally, to test the authenticity and accuracy of our ML tree, an NJ tree was constructed. The NJ tree (S3A Fig) and its topology (S3B Fig) showed the same results as those of the ML tree. Thus, the authenticity and accuracy of the ML tree constructed based on the *psbA-trnH* sequences is supported by conventional phylogeny-based identification methods, indicating that DNA barcoding using *psbA-trnH* sequences can be used to identify PR species.

### Character-based tests

A total of 14 variable positions at sites 22, 27, 65, 67, 103, 104,125, 127, 128, 129, 130, 132, 172, and 429 *psbA-trnH* sequences (S2 Table) were used to identify *Polygonatum* species. The combination of these 14 sites formed 11 compound CAs used to identify 22 species distributed in the sampling areas. Apart from *P*. *cyrtonema* (3), *P*. *cirrhifolium* (2), *P*. *odoratum* (2), *P*. *punctatum* (2), and *P*. *involucratum* (2), which had multiple corresponding CAs, the other 17 species were each associated with a single CA for species identification. Among these, only seven CAs were specific solely to their own species. *P*. *kingianum* (B17) and *P*. *sibiricum* (B26) had specific CAs, whereas three CAs (B5, B6, and B7) were found for *P*. *cyrtonema*. A single CA (B15) was observed for *P*. *involucratum*, while another (B24) was found for *P*. *punctatum*. Furthermore, two nonspecific CAs (B16 and B23) were also observed for *P*. *involucratum* and *P*. *punctatum*, but this association requires further confirmation.

These constructed CAs were then used to verify the identity of our 39 experimental samples (Tables 3 and 4). We found that our samples were differentiated in a manner that is similar to that observed using the nearest distance method corresponding to the “best close match.” Notably, the S11 and S22 CAs were not retrieved in this CA analysis, whereas they were identified as *D*. *longifolia* and *P*. *cyrtonema*, respectively, by the nearest distance method and phylogeny-based analysis. Thus, while we can precisely differentiate PR from other *Polygonatum* species and genera with this character-based method, discrepancies among the identification methods used in this study do exist.

## Discussion

Consumers purchase and consume PR based on its rhizome composition, which is largely identified on the basis of morphological characteristics. However, the rhizomes of other *Polygonatum* species and Asparagaceae genera are similar to those constituting PR, making them difficult to differentiate and easy to substitute into this herbal formulation. This can affect the biological effectiveness and safety of PR. Unfortunately, an efficient and accurate identification method has yet to be established for these types of medicinal rhizome formulations. In this study, we used three different methodologies to identify PR. Notably, all three enabled accurate identification of the PR components. Our results are the first to report an efficient approach, combining the three methods, for the characterization of PR, which can be used to discern the identity of PR components in formulations in the market.

### DNA barcoding-based identification of samples from different regions

DNA barcoding is an efficient and accurate method for true product identification that is not affected by the condition of the sample material. Barcoding gap, quantified as the difference between intraspecific and the smallest interspecific distance, has been used to evaluate DNA barcoding [49,50] and define new species [51], studies reported earlier showed that it is an artifact of insufficient sampling across taxa [52] and no distinct or sufficiently sized global barcoding gap exists [53]. Thus, it is useless and unworthiness for PR identification at species level due to inexistence of barcoding gap in PR samples in our study. This may because the number of sequences per species is small, and the study reported earlier supports this result [54]. *ITS2* and *psbA-trnH* as recommended DNA barcoding genes have been used to identify plants at the species level based on their high resolution [55] and fast evolutionary rate [56]. The *ITS2* sequence has been considered an ideal DNA barcoding sequence for species identification of fungi and higher plants [57], which revealed a 92.7% of resolution success rate at the species level [44, 58]. Among genes used for DNA barcoding in plants, *rpoB*, *rpoC1*, *matK*, *trnH-psbA*, *rbcL*, *ITS*, *accD*, *nhdJ*, *YCF5*, *UPA*, *atpF-atpH*, and *psbK-psbI*, *psbA-trnH* have demonstrated the best amplification success rates and species identification rates [59, 60]. However, the success rate of *ITS2* amplification is comparatively lower, and the sequencing of *cITS2* sequences is a little difficult [55], thereby limiting its application. In addition, a large number of insertions/deletions in the *psbA-trnH* sequence makes BLAST searches among species in different genera challenging. In this study, *ITS2* sequences amplified from samples using universal primers yielded polymorphic DNA bands, not as *psbA-trnH*. This fact may because universal primer of *ITS2* is not specific for PR and *Polygonatum* species. Study reported by Li [61] showed that *ITS2* regions was very low due to failure in PCR amplification for *Taxillus chinensis*, and this fact could support our result. As a matter of course, the reasons need further investigation. Although *ITS2* sequences were unsuitable for PR identification, the higher resolving power and accurate discrimination of PR obtained using the *psbA-trnH* sequence in this study indicates that this DNA barcoding system can be used to differentiate PR from other *Polygonatum* species and genera. Our results reflect similar findings reported earlier [62]. According to this method, our 39 samples were divided into five groups: *P*. *sibiricum* (7), *P*. *cyrtonema* (19), *P*. *kingianum* (9), *D*. *longifolia* (1), and undetermined (3). Notably, the undetermined groups could not be identified with the same GDs, and the “best close match” was observed for multiple species. This could be due to the low level of variation in the *psbA-trnH* sequence among these species. However, it is clear that the identity of the *P*. *punctatum* samples corresponded to their geographical origin.

### Phylogeny-based tests of *psbA-trnH* sequences

Phylogenetic tree construction can reveal interrelations among different species and can be used to judge the relationships between sample sequences and reference sequences based on their *psbA-trnH* sequence. These relationships can then be used to accurately identify the samples. Among the multiple phylogenetic trees constructed, the ML tree was considered to be the tree closest to the true tree for our samples. In fact, the ML tree based on the *psbA-trnH* sequences of our samples reflected the results based on the DNA barcoding system. In the ML tree, S30 was identified as *P*. *cyrtonema* and had a close relationship with *Heteropolygonatum roseolum*. Because three *psbA-trnH* sequences were downloaded from NCBI’s GenBank for *P*. *cyrtonema*, the samples identified as *P*. *sibiricum* were observed to have a close relationship with *P*. *cyrtonema* (KJ745888.1). This may again be due to the low level of variation in the *psbA-trnH* sequences of these two species. S8, S9, and S10 were also incompletely identified owing to their close relationships with numerous species. This indicates that the *psbA-trnH* sequences had a low identification efficiency for these species. The topology of the ML tree confirmed these results. Moreover, the NJ tree constructed in this study and its topology were also used to verify the reliability of the ML tree and the accuracy of our results. Our phylogeny-based tests revealed that DNA barcoding identification is an accurate method and can also be used to distinguish PR from adulterants or imitations.

### Character-based tests of *psbA-trnH* sequences

Character-based tests showed the same results as the DNA barcoding system and phylogeny-based methods. In this study, *P*. *cyrtonema*, *P*. *cirrhifolium*, *P*. *odoratum*, *P*. *punctatum*, and *P*. *involucratum* all had more than one CA, which could lead to mistakes in identifying PR. However, three CAs of *P*. *cyrtonema* were specific and could be used for identification. Thus, character-based tests of the *psbA-trnH* sequences can be further used to distinguish PR. Similar findings reported in other species [63] support our results. Compared with DNA barcoding-based and phylogeny-based methods, a character-based method has advantages for identifying species with lower variation in DNA barcoding. DNA barcoding-based and phylogeny-based methods are the main and universal methods, while phylogeny-based methods can identify components at not only the genus and family level but also the species level. Thus, combining all three methods would render our results more accurate.

### Application of DNA barcoding for the identification of PR

DNA barcoding has been used for identifying medicinal plants [64] and industrial quality assurance [65], such as for *Smithia conferta* Sm. [66], turmeric [67], *Crocus sativus* [68, 69], *Peucedanum praeruptorum* [70], radix astragali [71, 72], *Cinnamomum verum* [73], *Sabia parviflora* [74], *Valeriana jatamansi* [75], sandalwood [76], and *Hippophae* [30], which supports our findings. To date, it has been difficult to completely authenticate PR and its related products without relying on morphological characterization. Furthermore, the authenticity of raw materials is essential to guarantee product quality and consumer safety. DNA barcoding can efficiently and accurately identify products [23] regardless of their form. Thus, the method used in this study has immediate practical implications and can be quickly applied to molecularly identify PR.

However, for all *Polygonatum* species, DNA barcoding based on *psbA-trnH* sequences is limited due to lower genetic diversity, which might make inaccurate identification. Identification with more DNA barcodes or complete chloroplast genome and whole genome sequences would provide an effective method for *Polygonatum* species authentication. In addition, morphological characteristics of medicinal herbs plants can also be used to correctly classify species when they does not vary under different growing environment. Thus, more researches are needed to optimize and improve the method to molecularly identify *Polygonatum* species.

### Conclusions

A total of five species were identified in the 39 samples we analyzed from different growing regions: *P*. *sibiricum*, *P*. *cyrtonema*, *P*. *kingianum*, *D*. *longifolia*, and *P*. *punctatum*. Samples collected from four regions, S8−S11, were misidentified based on the morphological characteristics of their rhizomes. Our study indicates that this DNA barcoding identification method based on *psbA-trnH* sequences can efficiently and precisely differentiate PR from other species with the same rhizome characteristics. With this technology, PR quality can be preserved and improved for consumer consumption.

## Supporting information

S1 Fig Gel electrophoresis images of PCR products of *ITS2* (a) and *psbA-trnH* (b).
(TIF)

S2 Fig Genetic distance between sampling sequences and reference sequences shown in Tables 1 and 2. (TIF)

S3 Fig Phylogeny tree (a) and its topology (b) of neighbour-joining tree constructed based on *psbA-trnH* sequences in *Polygonatum* and outgroup.
(TIF)

S1 Table Details of reference sequences in *Polygonatum* and outgroup. (DOCX)

S2 Table Character based identification for polygonati rhizome species in *Polygonatum*. (DOCX)

S1 File Data of sequences of our samples. (XLSX)

## References

[1] Wujisguleng WJGL, Liu YJ, Long CL. Ethnobotanical review of food uses of *Polygonatum* (Convallariaceae) in China. Acta Soc Bot Pol. 2012;81(4):239–44. 10.5586/asbp.2012.045

[2] Jin JN. Verificine of polygonati rhizoma in connection with Buddhism and Taoism. National botany BBS in asia-pacific region; Nanjing2006. p. 323–9.

[3] Chinese PC. Pharmacopoeia of China. Beijing: China Medical Science Press; 2015. 306–7 p.

[4] Jiang QG, Lv YX, Dai WD, Miao XY, Zhong DW. Extraction and bioactivity of *Polygonatum* polysaccharides. Int J Biol Macromol. 2013;54:131–5. 10.1016/j.ijbiomac.2012.12.010 23246900

[5] Ma K, Huang XF, Kong LY. Steroidal saponins from *Polygonatum cyrtonema*. Chem Nat Compd^+^. 2013;49(5):888–91. 10.1007/s10600-013-0770-2

[6] Lan GS, Chen HX, Chen SH, Tian JG. Chemical composition and physicochemical properties of dietary fiber from *Polygonatum odoratum* as affected by different processing methods. Food Res Int. 2012;49(1):406–10. 10.1016/j.foodres.2012.07.047

[7] Gvazava LN, Kikoladze VS. Flavonoids from the plants *Polygonatum polyanthemum* and *P*. *glaberrimum*. Chem Nat Compd^+^. 2011;47(5):818–9. 10.1007/s10600-011-0072-5

[8] Li S. Compendium of Materia Medica. Shanghai: Shanghai science and technology press; 1993.

[9] Zhao XY, Li J. Chemical constituents of the genus *Polygonatum* and their role in medicinal treatment. Nat Prod Commun. 2015;10(4):683–8. 25973509

[10] Du L, Nong MN, Zhao JM, Peng XM, Zong SH, Zeng GF. *Polygonatum sibiricum* polysaccharide inhibits osteoporosis by promoting osteoblast formation and blocking osteoclastogenesis through Wnt/beta-catenin signalling pathway. Sci Rep-Uk. 2016;6 Artn 32261 10.1038/Srep32261 PMC4995504 27554324

[11] Guo S, Liu C, Liu S, Guan X, Guo L, Jia F, et al Streptomyces polygonati sp. nov., an endophytic actinomycete isolated from a root of *Polygonatum odoratum* (Mill.). International journal of systematic and evolutionary microbiology. 2016 10.1099/ijsem.0.000906 26790410

[12] Wang J, Lu CS, Liu DY, Xu YT, Zhu Y, Wu HH. Constituents from *Polygonatum sibiricum* and their inhibitions on the formation of advanced glycosylation end products. J Asian Nat Prod Res. 2016;18(7):697–704. 10.1080/10286020.2015.1135905 26841079

[13] Lu JM, Wang YF, Yan HL, Lin P, Gu W, Yu J. Antidiabetic effect of total saponins from *Polygonatum kingianum* in streptozotocin-induced daibetic rats. J Ethnopharmacol. 2016;179:291–300. 10.1016/j.jep.2015.12.057 26743227

[14] Yan HL, Lu JM, Wang YF, Gu W, Yang XX, Yu J. Intake of total saponins and polysaccharides from *Polygonatum kingianum* affects the gut microbiota in diabetic rats. Phytomedicine. 2017;26:45–54. 10.1016/j.phymed.2017.01.007 28257664

[15] Khan H, Saeed M, Muhammad N, Perviz S. Phytochemical analysis, antibacterial, and antifungal assessment of aerial parts of *Polygonatum verticillatum*. Toxicol Ind Health. 2016;32(5):841–7. 10.1177/0748233713512362 24311628

[16] Tai Y, Sun YM, Zou X, Pan Q, Lan YD, Huo Q, et al Effect of *Polygonatum odoratum* extract on human breast cancer MDA-MB-231 cell proliferation and apoptosis. Exp Ther Med. 2016;12(4):2681–7. 10.3892/etm.2016.3630 27698772 PMC5038215

[17] Peng MJ, Zhang YP, Shi SY. Separation of polar antioxidants from Rhizoma *Polygonatum* Odorati by high-speed counter-current chromatography with a hydrophilic solvent system. J Liq Chromatogr R T. 2016;39(4):171–7. 10.1080/10826076.2016.1141298

[18] Rhie YH, Lee SY, Park JH, Kim KS. Scarification and gibberellic acid affecting to dormancy breaking of Variegated Solomon's Seal (*Polygonatum odoratum* var. *pluriflorum* 'Variegatum'). Korean J Hortic Sci. 2014;32(3):296–302. 10.7235/hort.2014.13146

[19] Takagi H. Breaking of two types of dormancy in seeds of edible *Polygonatum macranthum*. J Jpn Soc Hortic Sci. 2001;70(4):424–30.

[20] Hebert PDN, Cywinska A, Ball SL, DeWaard JR. Biological identifications through DNA barcodes. P Roy Soc B-Biol Sci. 2003;270(1512):313–21. 10.1098/rspb.2002.2218 12614582 PMC1691236

[21] Xu MNA, Heidmarsson S, Thorsteinsdottir M, Eiriksson FF, Omarsdottir S, Olafsdottir ES. DNA barcoding and LC-MS metabolite profiling of the lichen-forming genus *Melanelia*: Specimen identification and discrimination focusing on Icelandic taxa. Plos One. 2017;12(5). doi: ARTN e0178012 10.1371/journal.pone.0178012 PMC5443556 28542495

[22] Oberlies N. How do you know that material is what it says it is? DNA barcoding for the taxonomic identification of fungi. Toxicol Lett. 2017;280:S39–S40.

[23] Raja HA, Baker TR, Little JG, Oberlies NH. DNA barcoding for identification of consumer-relevant mushrooms: A partial solution for product certification? Food Chem. 2017;214:383–92. 10.1016/j.foodchem.2016.07.052 27507489

[24] Tang YL, Wu YS, Huang RS, Chao NX, Liu Y, Xu P, et al Molecular identification of *Uncaria* (Gouteng) through DNA barcoding. Chin Med-Uk. 2016;11. doi: ARTN 3 10.1186/s13020-015-0072-7. PMC4739391 26843891

[25] Yu M, Liu K, Zhou L, Zhao L, Liu SQ. Testing three proposed DNA barcodes for the wood identification of *Dalbergia odorifera* T. Chen and *Dalbergia tonkinensis* Prain. Holzforschung. 2016;70(2):127–36. 10.1515/hf-2014-0234

[26] Nithaniyal S, Parani M. Evaluation of chloroplast and nuclear DNA barcodes for species identification in *Terminalia* L. Biochem Syst Ecol. 2016;68:223–9.

[27] Han YW, Duan D, Ma XF, Jia Y, Liu ZL, Zhao GF, et al Efficient identification of the forest tree species in Aceraceae using DNA barcodes. Front Plant Sci. 2016;7. doi: ARTN 1707 10.3389/fpls.2016.01707. PMC5110567 27899929

[28] Enan MR, Ahmed A. Cultivar-level phylogeny using chloroplast DNA barcode *psbK-psbI* spacers for identification of Emirati date palm (*Phoenix dactylifera* L.) varieties. Genet Mol Res. 2016;15(3). doi: ARTN 15038470 10.4238/gmr.15038470. 27525916

[29] Mahadani P, Sharma GD, Ghosh SK. Identification of ethnomedicinal plants (Rauvolfioideae: Apocynaceae) through DNA barcoding from northeast India. Pharmacogn Mag. 2013;9(35):255–63. 10.4103/0973-1296.113284 23930011 PMC3732430

[30] Liu Y, Xiang L, Zhang Y, Lai XR, Xiong C, Li JJ, et al DNA barcoding based identification of *Hippophae* species and authentication of commercial products by high resolution melting analysis. Food Chem. 2018;242:62–7. 10.1016/j.foodchem.2017.09.040 29037736

[31] Zhang DQ, Mo XC, Xiang JY, Zhou N. Molecular identification of original plants of fritillariae cirrhosae bulbus, a tradtional Chinese medicine (Tcm) using plant DNA barcoding. Afr J Tradit Complem. 2016;13(6):74–82. 10.21010/ajtcam.v13i6.12 28480363 PMC5412205

[32] Lv TW, Teng RD, Shao QS, Wang HZ, Zhang WS, Li MY, et al DNA barcodes for the identification of *Anoectochilus roxburghii* and its adulterants. Planta. 2015;242(5):1167–74. 10.1007/s00425-015-2353-x 26105653

[33] Moon BC, Kim WJ, Ji Y, Lee YM, Kang YM, Choi G. Molecular identification of the traditional herbal medicines, Arisaematis Rhizoma and Pinelliae Tuber, and common adulterants via universal DNA barcode sequences. Genet Mol Res. 2016;15(1). UNSP gmr.15017064 10.4238/gmr.15017064. 26909979

[34] Kim WJ, Ji Y, Choi G, Kang YM, Yang S, Moon BC. Molecular identification and phylogenetic analysis of important medicinal plant species in genus *Paeonia* based on *rDNA-ITS*, *matK*, and *rbcL* DNA barcode sequences. Genet Mol Res. 2016;15(3). 10.4238/gmr.15038472 27525917

[35] Hirsch AM, Moraes DC. Identification of the south American medicinal plant *Baccharis genistelloides* ("carqueja") using DNA barcodes. Abstr Pap Am Chem S. 2014;248.

[36] Chen XC, Xiang L, Shi LC, Li G, Yao H, Han JP, et al Identification of crude drugs in the Japanese pharmacopoeia using a DNA barcoding system. Sci Rep-Uk. 2017;7 ARTN 42325 10.1038/srep42325 PMC5301229 28186159

[37] Song XN, Li YP, Xu GJ, Liu CS, Liu Y, Zhang XQ, et al Identification of Notoginseng powder based on similarity to "DNA Barcoding Core-genotype''. Mitochondrial DNA A. 2017;28(3):355–7. 10.3109/19401736.2015.1122777 26714125

[38] Song M, Dong GQ, Zhang YQ, Liu X, Sun W. Identification of processed Chinese medicinal materials using DNA mini-barcoding. Chin J Nat Medicines. 2017;15(7):481–6. 10.1016/S1875-5364(17)30073-0 28807221

[39] Nithaniyal S, Vassou SL, Poovitha S, Raju B, Parani M. Identification of species adulteration in traded medicinal plant raw drugs using DNA barcoding. Genome. 2017;60(2):139–46. 10.1139/gen-2015-0225 28067539

[40] Yao H, Song JY, Ma XY, Liu C, Li Y, Xu HX, et al Identification of *Dendrobium* species by a candidate DNA barcode sequence: The chloroplast psbA-trnH intergenic region. Planta Med. 2009;75(6):667–9. 10.1055/s-0029-1185385 19235685

[41] Song JY, Yao H, Li Y, Li XW, Lin YL, Liu C, et al Authentication of the family Polygonaceae in Chinese pharmacopoeia by DNA barcoding technique. J Ethnopharmacol. 2009;124(3):434–9. 10.1016/j.jep.2009.05.042 19505556

[42] Pang XH, Chen SL. Using DNA barcodes to identify Rosaceae. Planta Med. 2009;75(4):417–.

[43] Luo K, Chen SL, Chen KL, Song JY, Yao H. Application of DNA barcoding to the medicinal plants of the Araceae family. Planta Med. 2009;75(4):416–.

[44] Gao T, Chen SL. Authentication of the medicinal plants in Fabaceae by DNA barcoding technique. Planta Med. 2009;75(4):417–.

[45] He LH, Zhao Y, Chen MJ, Pan YJ. An efficient method for DNA extraction from compost. Acta microbiologica Sinica. 2006;46(1):162–5. 16579488

[46] Ross HA, Murugan S, Li WLS. Testing the reliability of genetic methods of species identification via simulation. Systematic Biol. 2008;57(2):216–30. 10.1080/10635150802032990 18398767

[47] Lowenstein JH, Amato G, Kolokotronis SO. The real maccoyii: identifying tuna sushi with DNA barcodes—contrasting characteristic attributes and genetic distances. PloS one. 2009;4(11):e7866 10.1371/journal.pone.0007866 19924239 PMC2773415

[48] Meier R, Zhang G, Ali F. The use of mean instead of smallest interspecific distances exaggerates the size of the “barcoding gap” and leads to misidentification. Systematic Biol. 2008;57(5):809–13. 10.1080/10635150802406343 18853366

[49] Yang Z, Rannala B. Bayesian species identification under the multispecies coalescent provides significant improvements to DNA barcoding analyses. Mol Ecol. 2017;26(11):3028–36. 10.1111/mec.14093 28281309

[50] Puillandre N, Lambert A, Brouillet S, Achaz G. ABGD, Automatic Barcode Gap Discovery for primary species delimitation. Mol Ecol. 2012;21(8):1864–77. 10.1111/j.1365-294X.2011.05239.x 21883587

[51] Hebert PD, Stoeckle MY, Zemlak TS, Francis CM. Identification of birds through DNA barcodes. Plos Biology. 2004;2(10):e312 10.1371/journal.pbio.0020312 15455034 PMC518999

[52] Wiemers M, Fiedler K. Does the DNA barcoding gap exist?–a case study in blue butterflies (Lepidoptera: Lycaenidae). Front Zool. 2007;4(1):8. 17343734 10.1186/1742-9994-4-8 PMC1838910

[53] Kvist S. Does a global DNA barcoding gap exist in Annelida? Mitochondrial DNA A. 2016;27(3):2241–52. 10.3109/19401736.2014.984166 25431824

[54] Chen J, Zhao J, Erickson DL, Xia N, Kress WJ. Testing DNA barcodes in closely related species of *Curcuma* (Zingiberaceae) from Myanmar and China. Mol Ecol Resour. 2015;15(2):337–48. 10.1111/1755-0998.12319 25158042

[55] Ren BQ, Xiang XG, Chen ZD. Species identification of *Alnus* (Betulaceae) using nrDNA and cpDNA genetic markers. Mol Ecol Resour. 2010;10(4):594–605. 10.1111/j.1755-0998.2009.02815.x 21565064

[56] Shaw J, Lickey EB, Beck JT, Farmer SB, Liu W, Miller J, et al The tortoise and the hare II: relative utility of 21 noncoding chloroplast DNA sequences for phylogenetic analysis. Am J Bot. 2005;92(1):142–66. 10.3732/ajb.92.1.142 21652394

[57] Yao H, Song JY, Liu C, Luo K, Han JP, Li Y, et al Use of *ITS2* region as the universal DNA barcode for plants and animals. PloS one. 2010;5(10):-. 10.1371/journal.pone.0013102 PMC2948509 20957043

[58] Chen S, Yao H, Han J, Liu C, Song J, Shi L, et al Validation of the *ITS2* region as a novel DNA barcode for identifying medicinal plant species. Plos One. 2010;5(1):e8613 10.1371/journal.pone.0008613 20062805 PMC2799520

[59] Kress WJ, Erickson DL. A two-locus global DNA barcode for land plants: the coding *rbcL* gene complements the non-coding *trnH-psbA* spacer region. Plos One. 2007;2(6):e508 10.1371/journal.pone.0000508 17551588 PMC1876818

[60] Fazekas AJ, Burgess KS, Kesanakurti PR, Graham SW, Newmaster SG, Husband BC, et al Multiple multilocus DNA barcodes from the plastid genome discriminate plant species equally well. Plos One. 2008;3(7):e2802 10.1371/journal.pone.0002802 18665273 PMC2475660

[61] Li YH, Jinlan R, Chen SL, Song JY, Luo K, Dong L, et al Authentication of *Taxillus chinensis* using DNA barcoding technique. J Med Plants Res. 2010;4(24):2706–9.

[62] Yang P, Zhou H, Xin T, Ma S, Duan B, Yao H. Identification study of DNA barcode sequences in the medicinal plants of *Polygonatum*. World Chinese Medicine. 2015;10(8):1173–6.

[63] Zou SM, Li Q. Pay attention to the overlooked cryptic diversity in existing barcoding data: the case of Mollusca with character-based DNA barcoding. Mar Biotechnol. 2016;18(3):327–35. 10.1007/s10126-016-9692-x 26899167

[64] Gao ZT, Liu Y, Wang XY, Song JY, Chen SL, Ragupathy S, et al Derivative technology of DNA barcoding (nucleotide signature and SNP double peak methods) detects adulterants and substitution in Chinese patent medicines. Sci Rep-Uk. 2017;7. 10.1038/s41598-017-05892-y PMC5517575 28724933

[65] Sgamma T, Lockie-Williams C, Kreuzer M, Williams S, Scheyhing U, Koch E, et al DNA barcoding for industrial quality assurance. Planta Med. 2017;83(14–15):1117–29. 10.1055/s-0043-113448 28662530

[66] Umdale SD, Kshirsagar PR, Lekhak MM, Gaikwad NB. Molecular authentication of the traditional medicinal plant "Lakshman Booti" (*Smithia conferta* Sm.) and its adulterants through DNA barcoding. Pharmacogn Mag. 2017;13(50):S224–S9. 28808384 10.4103/pm.pm_499_16 PMC5538158

[67] Parvathy VA, Swetha VP, Sheeja TE, Sasikumar B. Detection of plant-based adulterants in turmeric powder using DNA barcoding. Pharm Biol. 2015;53(12):1774–9. 10.3109/13880209.2015.1005756 25853978

[68] Huang WJ, Li FF, Liu YJ, Long CL. Identification of *Crocus sativus* and its adulterants from Chinese markets by using DNA barcoding technique. Iran J Biotechnol. 2015;13(1):36–42. ARTN e1034 10.15171/ijb.1034 28959279 PMC5434985

[69] Huang W, Li F, Liu Y, Long C. Identification of *Crocus sativus* (Iridaceae) and its adulterants by using DNA barcoding technique. Planta Med. 2014;80(10):846–.

[70] Zhou J, Wang WC, Liu MQ, Liu ZW. Molecular authentication of the traditional medicinal plant *Peucedanum praeruptorum* and its substitutes and adulterants by DNA—barcoding technique. Pharmacogn Mag. 2014;10(40):385–90. 10.4103/0973-1296.141754 25422535 PMC4239712

[71] Zheng SH, Liu DW, Ren WG, Fu J, Huang LF, Chen SL. Integrated analysis for identifying *Radix Astragali* and its adulterants based on DNA barcoding. Evid-Based Compl Alt. 2014 Artn 843923 10.1099/ijsem.0.000906 PMC4160622 25246939

[72] Guo HY, Wang WW, Yang N, Guo BL, Zhang S, Yang RJ, et al DNA barcoding provides distinction between *Radix Astragali* and its adulterants. Sci China Life Sci. 2010;53(8):992–9. 10.1007/s11427-010-4044-y 20821298

[73] Swetha VP, Parvathy VA, Sheeja TE, Sasikumar B. DNA barcoding for discriminating the economically important *Cinnamomum verum* from its Adulterants. Food Biotechnol. 2014; 28(3):183–94. 10.1080/08905436.2014.931239

[74] Sui XY, Huang YA, Tan Y, Guo Y, Long CL. Molecular authentication of the ethnomedicinal plant *Sabia parviflora* and its adulterants by DNA barcoding technique. Planta Med. 2011;77(5):492–6. 10.1055/s-0030-1250468 20979018

[75] Yang Y, Zhai YH, Liu T, Zhang FM, Ji YH. Detection of *Valeriana jatamansi* as an adulterant of medicinal *Paris* by length variation of chloroplast *psbA-trnH* region. Planta Med. 2011;77(1):87–91. 10.1055/s-0030-1250072 20597045

[76] Dev SA, Muralidharan EM, Sujanapal P, Balasundaran M. Identification of market adulterants in east Indian sandalwood using DNA barcoding. Ann Forest Sci. 2014;71(4):517–22. 10.1007/s13595-013-0354-0

