# Supplementary figures and images for: DNA barcoding for the efficient and accurate identification of medicinal polygonati rhizoma in China
Source: PLoS One. 2018 Jul 18;13(7):e0201015. doi: 10.1371/journal.pone.0201015 (PMC6051646; doi:10.1371/journal.pone.0201015)

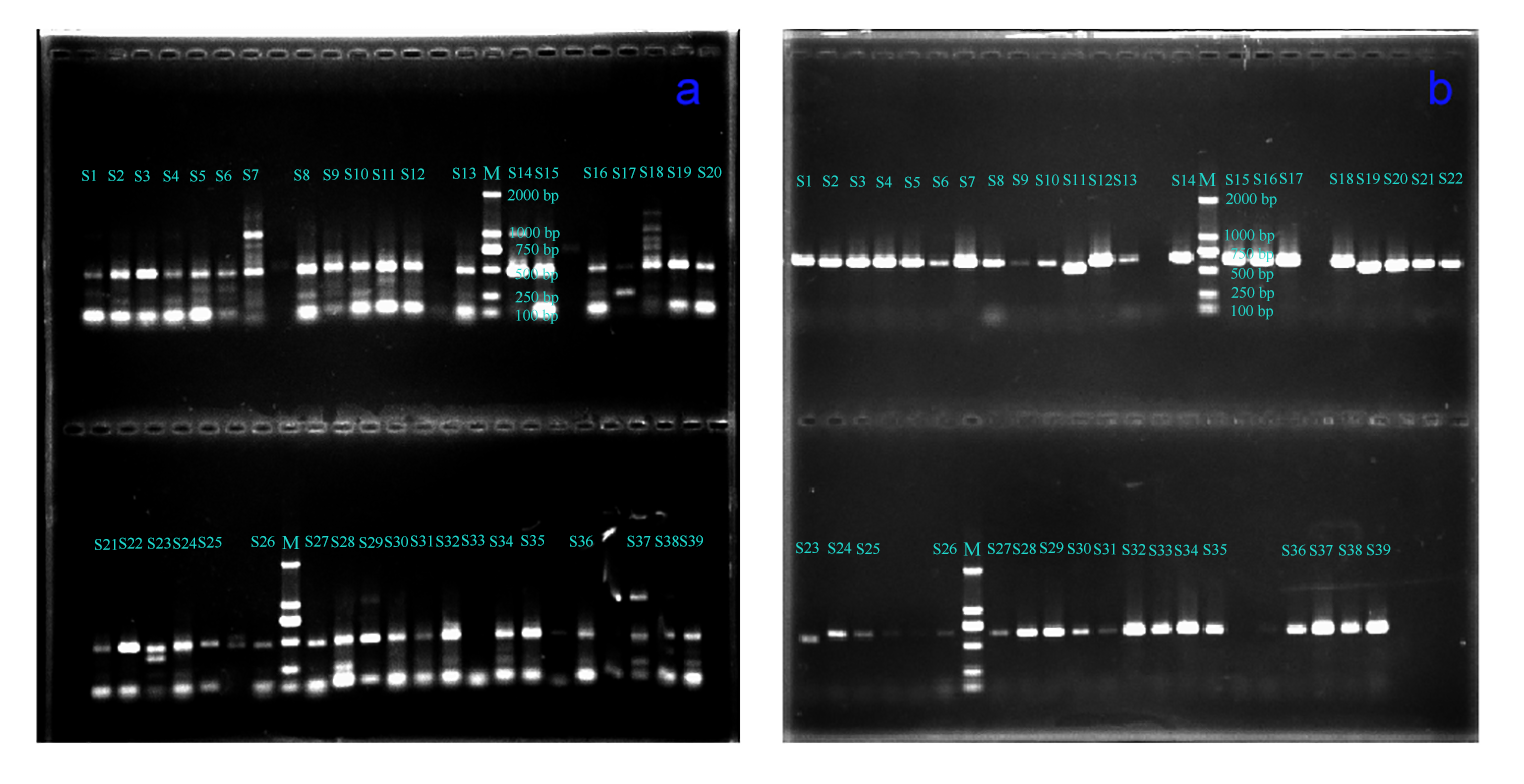

Supplement: S1 Fig — Gel electrophoresis images of PCR products of ITS2 (a) and psbA-trnH (b). (TIF) [file pone.0201015.s001.tif]

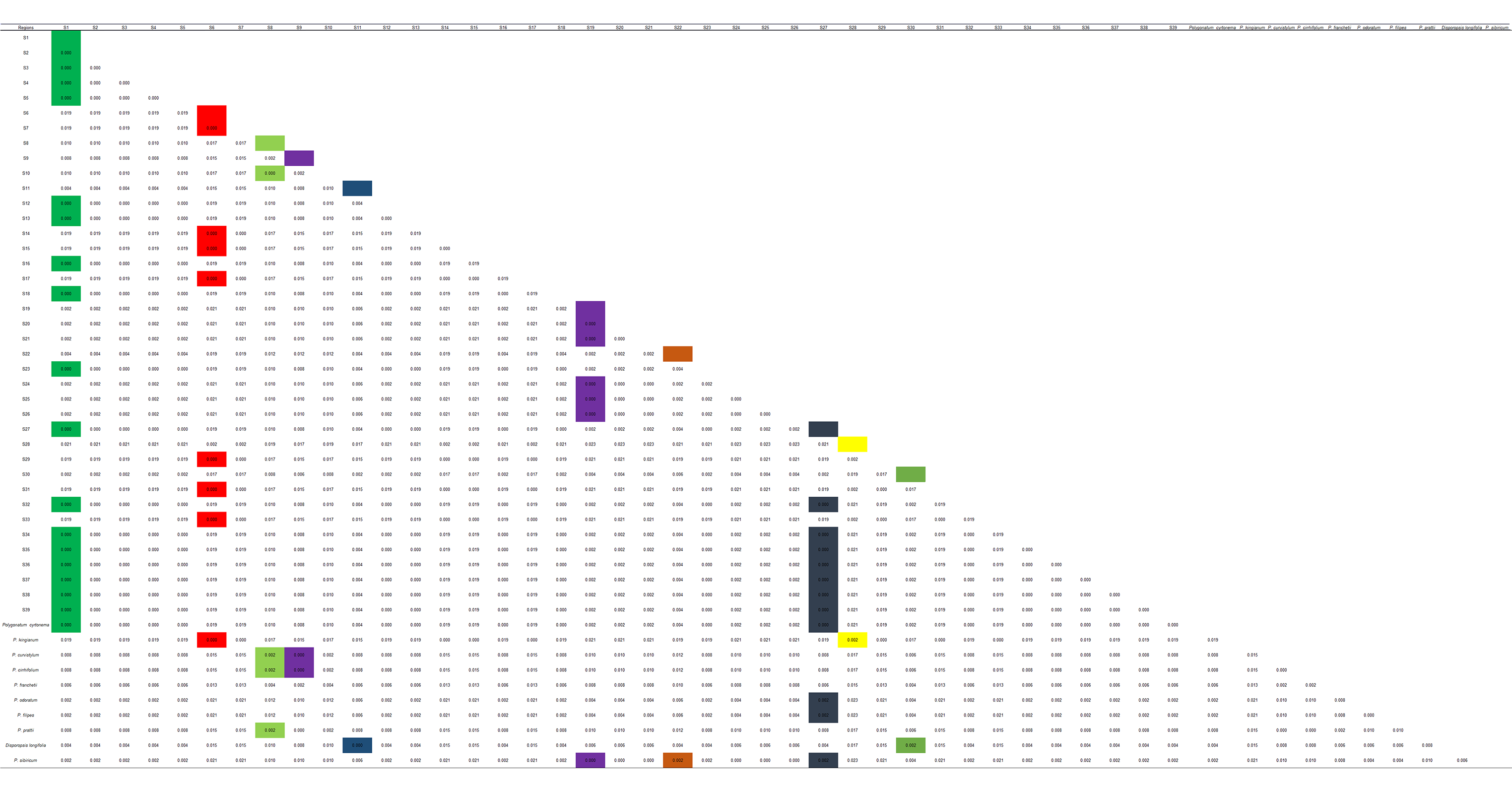

Supplement: S2 Fig — (TIF) [file pone.0201015.s002.tif]

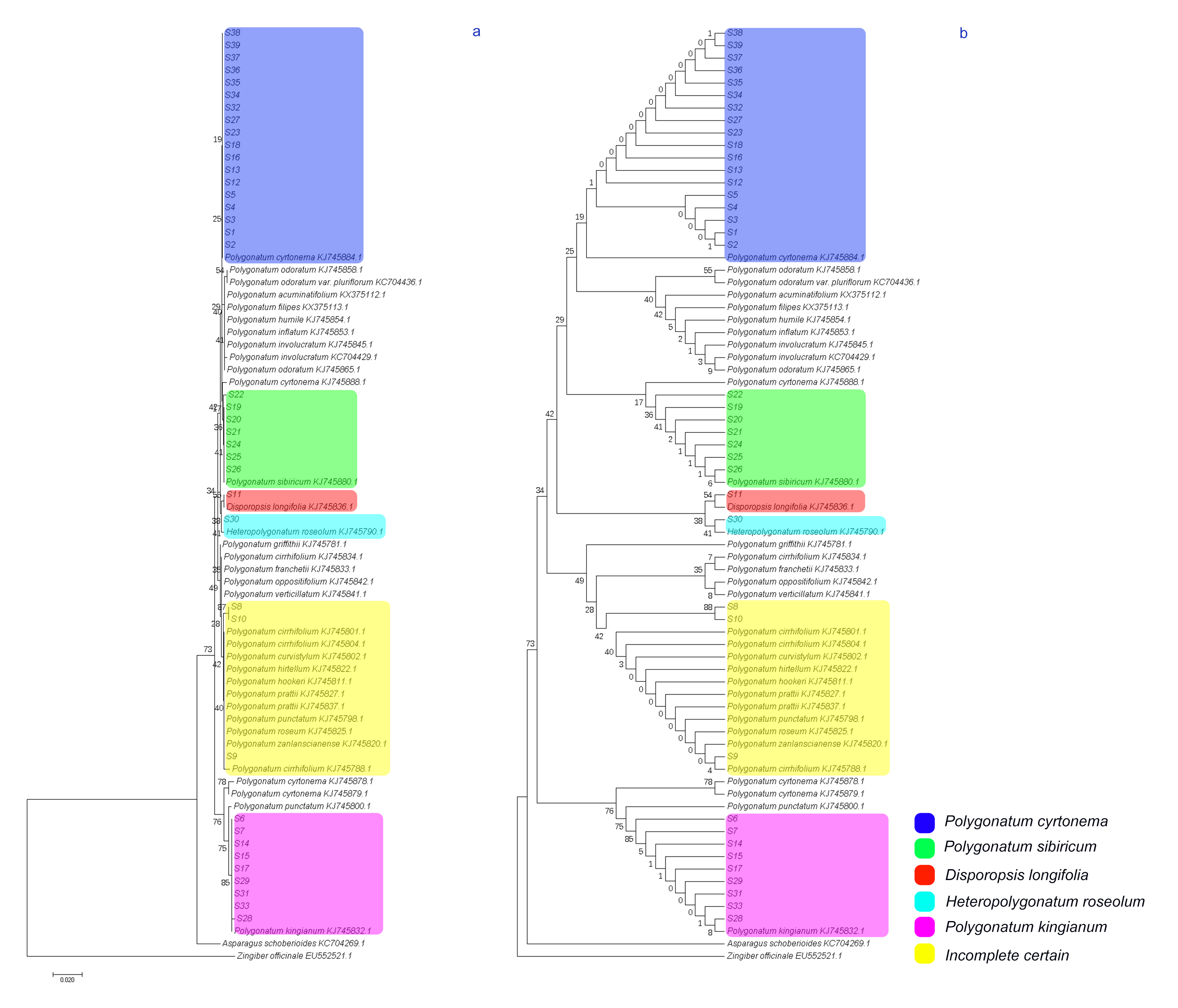

Supplement: S3 Fig — Phylogeny tree (a) and its topology (b) of neighbour-joining tree constructed based on psbA-trnH sequences in Polygonatum and outgroup. (TIF) [file pone.0201015.s003.tif]
