# Supplementary material for: DNA barcoding for the efficient and accurate identification of medicinal polygonati rhizoma in China
Source: PLoS One. 2018 Jul 18;13(7):e0201015. doi: 10.1371/journal.pone.0201015 (PMC6051646; doi:10.1371/journal.pone.0201015)
Supplement: S1 Table — (DOCX) [file pone.0201015.s004.docx]

**Table S1** **Details of reference sequences in *Polygonatum* and outgroup.**

| **Serial number** | **Species** | **Accession number** |
| --- | --- | --- |
| F1 | *Polygonatum acuminatifolium* | KX375112.1 |
| F2 | *Polygonatum cirrhifolium* | KJ745788.1 |
| F3 | *Polygonatum cirrhifolium* | KJ745801.1 |
| F4 | *Polygonatum cirrhifolium* | KJ745804.1 |
| F5 | *Polygonatum cirrhifolium* | KJ745834.1 |
| F6 | *Polygonatum curvistylum* | KJ745802.1 |
| F7 | *Polygonatum cyrtonema* | KJ745878.1 |
| F8 | *Polygonatum cyrtonema* | *KJ745879.1* |
| F9 | *Polygonatum cyrtonema* | KJ745884.1 |
| F10 | *Polygonatum cyrtonema* | KJ745888.1 |
| F11 | *Polygonatum filipes* | KX375113.1 |
| F12 | *Polygonatum franchetii* | KJ745833.1 |
| F13 | *Polygonatum griffithii* | KJ745781.1 |
| F14 | *Polygonatum hirtellum* | KJ745822.1 |
| F15 | *Polygonatum hookeri* | KJ745811.1 |
| F16 | *Polygonatum humile* | KJ745854.1 |
| F17 | *Polygonatum inflatum* | KJ745853.1 |
| F18 | *Polygonatum involucratum* | KC704429.1 |
| F19 | *Polygonatum involucratum* | KJ745845.1 |
| F20 | *Polygonatum kingianum* | KJ745832.1 |
| F21 | *Polygonatum odoratum* | KJ745858.1 |
| F22 | *Polygonatum odoratum* | KJ745865.1 |
| F23 | *Polygonatum odoratum* var *pluriflorum* | KC704436.1 |
| F24 | *Polygonatum oppositifolium* | KJ745842.1 |
| F25 | *Polygonatum prattii* | KJ745827.1 |
| F26 | *Polygonatum prattii* | KJ745837.1 |
| F27 | *Polygonatum punctatum* | KJ745798.1 |
| F28 | *Polygonatum punctatum* | KJ745800.1 |
| F29 | *Polygonatum roseum* | KJ745825.1 |
| F30 | *Polygonatum sibiricum* | KJ745880.1 |
| F31 | *Polygonatum verticillatum* | KJ745841.1 |
| F32 | *Polygonatum zanlanscianense* | KJ745820.1 |
| F33 | *Disporopsis longifolia* | KJ745836.1 |
| F34 | *Asparagus schoberioides* | KC704269.1 |
| F35 | *Heteropolygonatum roseolum* | KJ745790.1 |
| F36 | *Zingiber officinale* | EU552521.1 |
