# Supplementary material for: DNA barcoding for the efficient and accurate identification of medicinal polygonati rhizoma in China
Source: PLoS One. 2018 Jul 18;13(7):e0201015. doi: 10.1371/journal.pone.0201015 (PMC6051646; doi:10.1371/journal.pone.0201015)
Supplement: S2 Table — (DOCX) [file pone.0201015.s005.docx]

**Table S2** **Character based identification for** **Polygonati rhizome species in *Polygonatum*.**

| **Menu listing** | **Character positions**  **(****22, 27, 65, 67, 103, 104,125, 127, 128, 129, 130, 132, 172, 429)** | **Species** |
| --- | --- | --- |
| B1 | CCCGTCGTTTCTAA | *Polygonatum acuminatifolium* |
| B2 | CGCACTGTTTCTTA | *Polygonatum cirrhifolium* |
| B3 | CGCGCTGTTTCTTA | *Polygonatum cirrhifolium* |
| B4 | CGCACTGTTTCTTA | *Polygonatum curvistylum* |
| B5 | CGAGTCAGAAACTC | *Polygonatum cyrtonema* |
| B6 | CGCGTCAGAAACTA | *Polygonatum cyrtonema* |
| B7 | CGCGTCGTTTCTTA | *Polygonatum cyrtonema* |
| B8 | CCCGTCGTTTCTAA | *Polygonatum filipes* |
| B9 | CGCGCTGTTTCTTA | *Polygonatum franchetii* |
| B10 | CGCGCTGTTTCTTA | *Polygonatum griffithii* |
| B11 | CGCACTGTTTCTTA | *Polygonatum hirtellum* |
| B12 | CGCACTGTTTCTTA | *Polygonatum hookeri* |
| B13 | CCCGTCGTTTCTAA | *Polygonatum humile* |
| B14 | CCCGTCGTTTCTAA | *Polygonatum inflatum* |
| B15 | GCCGTCGTTTCTAA | *Polygonatum involucratum* |
| B16 | CCCGTCGTTTCTAA | *Polygonatum involucratum* |
| B17 | CGCGCTAGAAACTC | *Polygonatum kingianum* |
| B18 | CCCGTCGTTTCTAC | *Polygonatum odoratum* |
| B19 | CCCGTCGTTTCTAA | *Polygonatum odoratum* |
| B20 | CCCGTCGTTTCTAC | *Polygonatum odoratum* var. *pluriflorum* |
| B21 | CGCGCTGTTTCTTA | *Polygonatum oppositifolium* |
| B22 | CGCACTGTTTCTTA | *Polygonatum prattii* |
| B23 | CGCACTGTTTCTTA | *Polygonatum* *punctatum* |
| B24 | CGCACTAGAAACTA | *Polygonatum punctatum* |
| B25 | CGCACTGTTTCTTA | *Polygonatum roseum* |
| B26 | CGCGTCGTTTCTCA | *Polygonatum sibiricum* |
| B27 | CGCGCTGTTTCTTA | *Polygonatum verticillatum* |
| B28 | CGCACTGTTTCTTA | *Polygonatum zanlanscianense* |
